# Quercetin alleviates cyclophosphamide-induced premature ovarian insufficiency in mice by reducing mitochondrial oxidative stress and pyroptosis in granulosa cells

**DOI:** 10.1186/s13048-022-01080-3

**Published:** 2022-12-27

**Authors:** Yun Chen, Ying Zhao, Chenyun Miao, Liuqing Yang, Ruye Wang, Bixia Chen, Qin Zhang

**Affiliations:** Department of TCM Gynecology, Hangzhou TCM Hospital Affiliated to Zhejiang Chinese Medical University, 453 Tiyuchang Road, Xihu District, Hangzhou, 310007 Zhejiang Province China

**Keywords:** Premature ovarian insufficiency, Cyclophosphamide, Quercetin, Mitochondria, Pyroptosis

## Abstract

**Background:**

Exposure to cyclophosphamide (CTX) induces premature ovarian insufficiency (POI). Quercetin is a natural flavonoid that exhibits anti-inflammatory and antioxidant properties, and its antioxidant activity is correlated with POI. However, the mechanism underlying its protective role in CTX-induced ovarian dysfunction is unclear. This study aimed to explore whether quercetin can protect ovarian reserves by activating mitochondrial biogenesis and inhibiting pyroptosis.

**Methods:**

Thirty-six female C57BL/6 mice were randomly subdivided into six groups. Except for the control group, all groups were injected with 90 mg/kg CTX to establish a POI model and further treated with coenzyme 10 or various doses of quercetin. The mice were sacrificed 48 h after 10 IU pregnant mare serum gonadotropin was injected four weeks after treatments. We used enzyme-linked immunosorbent assays to detect serum hormone expression and light and transmission electron microscopy to assess ovarian tissue morphology and mitochondria. Additionally, we tested oxidant and antioxidant levels in ovarian tissues and mitochondrial function in granulosa cells (GCs). The expression of mitochondrial biogenesis and pyroptosis-related proteins and mRNA was analyzed using western blotting and RT-qPCR.

**Results:**

Quercetin elevated serum anti-Müllerian hormone, estradiol, and progesterone levels, decreased serum follicle-stimulating hormone and luteinizing hormone levels, and alleviated ovarian pathology. It reduced the mitochondrial DNA content and mitochondrial membrane potential. Furthermore, it upregulated ATP levels and the mRNA and protein expression of peroxisome proliferator-activated receptor gamma coactivator 1-alpha (PGC1α), mitochondrial transcription factor A, and superoxide dismutase 2. In addition, it suppressed NOD-like receptor pyrin domain containing 3, caspase-1, interleukin-1β, and gasdermin D levels in the GCs of POI mice.

**Conclusions:**

Quercetin protected the ovarian reserve from CTX-induced ovarian damage by reversing mitochondrial dysfunction and activating mitochondrial biogenesis via the PGC1-α pathway. Moreover, quercetin may improve ovarian functions by downregulating pyroptosis in the CTX-induced POI model. Thus, quercetin can be considered a potential agent for treating POI.

## Background

With the aging of the population, the number of people with tumors has increased globally, including in women of reproductive age. Many chemotherapeutic drugs used for cancer treatment often exhibit serious gonadal-toxic effects [1]. Exposure to chemotherapy is regarded as a specific risk factor for premature ovarian insufficiency (POI), resulting in menstrual disorders, metabolic abnormalities, and infertility [2]. Although chemotherapeutic drugs inhibit the growth and spread of tumors, the quality of life and reproductive ability of female patients are susceptible to adverse effects.

Cyclophosphamide (CTX), a chemotherapeutic drug commonly used to treat various forms of cancer, causes apparent damage to the ovaries with dose-dependent effects [3, 4]. Recent studies have demonstrated that CTX-induced ovary damage is associated with the facilitation of granulosa cell (GC) apoptosis [5]. GCs, the largest cell group in the ovary, are responsible for the synthesis of estrogen and progesterone and are closely involved in follicle development, ovulation, and atresia [6]. The growth rate of GCs can affect the transcriptional activity of oocytes, which is regulated by the gap junctions between them [7, 8]. An experimental study confirmed that the apoptosis level of GCs in a POI model group was substantially higher than that in the normal group [9]. In contrast, increased inflammatory factor levels and decreased antioxidant capacity play a fundamental role in CTX-induced ovary damage [10, 11].

Reactive oxygen species (ROS) are a double-edged sword. Certain levels of ROS are essential for typical female physiological processes, such as oocyte maturation and fertilization, embryo development, and pregnancy [12]. Oxidative stress (OS), resulting from the excessive generation of ROS and defective antioxidant defense mechanisms, is believed to be the primary cause of GC apoptosis [13]. The toxic effects of CTX are due to its active metabolites, which can hinder DNA synthesis and interfere with the antioxidant defense mechanisms of the ovary, resulting in the overproduction of the ROS malondialdehyde (MDA) [14].

Mitochondria are the main site of ROS production; hence, they are a primary target of ROS, which can lead to fatal consequences [15]. For example, hydroxyl radicals, a common ROS, can damage mitochondrial DNA (mtDNA) and oxidize mitochondrial proteins and lipids [16]. Moreover, the release of ROS and mtDNA is a critical upstream event that triggers the activation of NOD-like receptor pyrin domain containing 3 (NLRP3) inflammasome. NLRP3 induces a rapid, inflammatory form of programmed cell death called pyroptosis, accompanied by enhanced secretion of interleukin (IL)-1β and IL-18 [17]. Previous studies have shown that oxidative stress activates inflammasomes in oocytes and GCs [18, 19].

However, there are no widely recognized protective agents. Thus, a new approach is required to prevent ovarian damage from chemotherapy drugs, preserve future fertility, maintain female endocrine function, and avoid the long-term complications of POI.

Quercetin is a natural flavonoid that is widely found in many fruits and plants, including tea, onions, apples, and kale [20]. Owing to its high solubility and bioavailability, it has been widely studied for its therapeutic and preventive effects on various diseases [21]. Several studies have demonstrated its excellent safety and efficacy in multiple cancers, Alzheimer’s disease, and metabolic diseases [22–24]. Quercetin has potent antioxidant and anti-inflammatory properties [25]. Interestingly, several in vitro and in vivo studies have reported the correlation between the antioxidant activity of quercetin and POI [26, 27]. However, the mechanism through which quercetin inhibits OS in CTX-induced ovarian dysfunction remains unclear.

This study used a CTX-induced POI mouse model to explore whether quercetin could improve ovarian function by suppressing OS and activating mitochondrial biogenesis. Furthermore, we explored the possible anti-pyroptosis properties of quercetin in alleviating ovarian damage following CTX exposure.

## Results

### Quercetin lowered follicle-stimulating hormone (FSH) and luteinizing hormone (LH) levels and increased anti-Müllerian hormone (AMH), estradiol (E2), and progesterone (P) expression

**Fig. 1:**
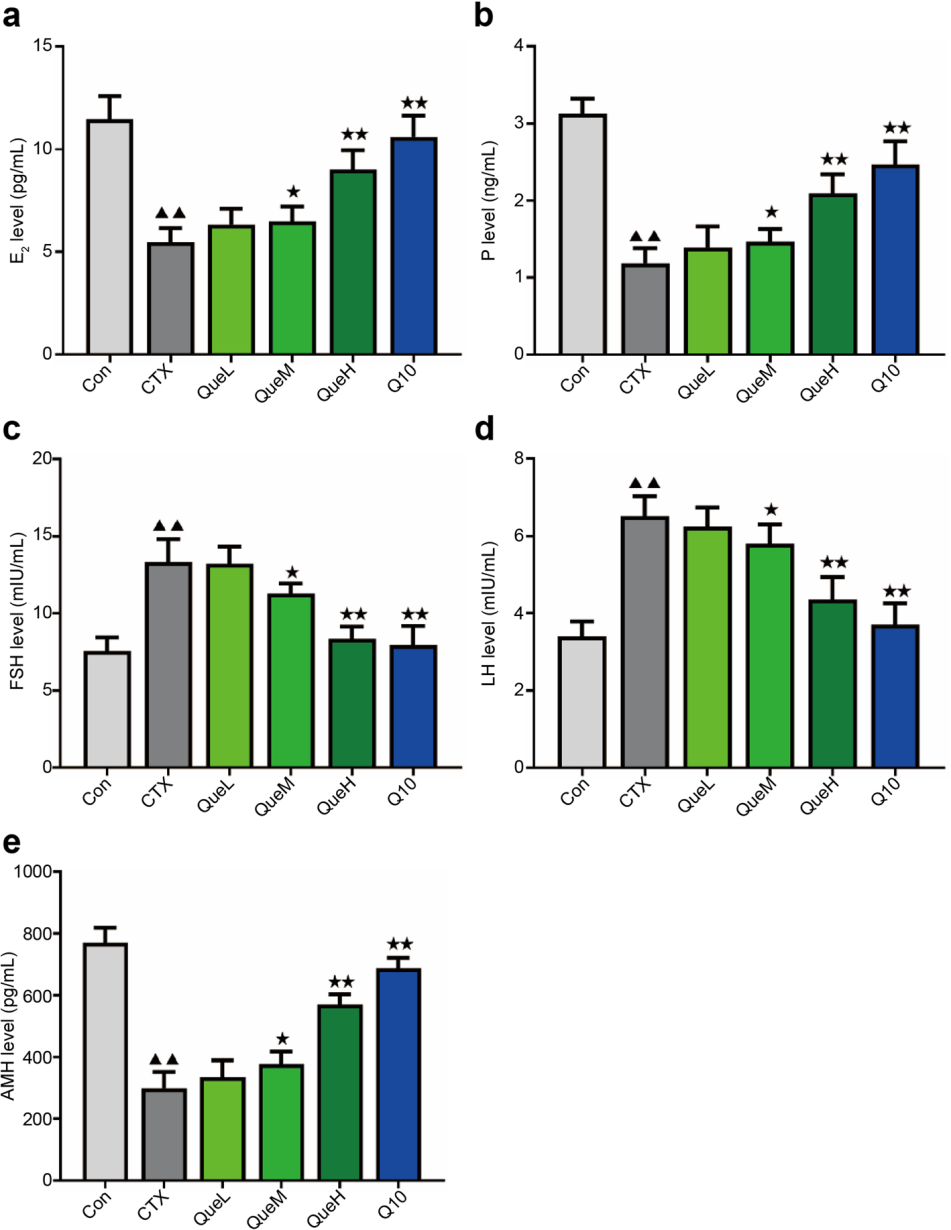
ELISA detection of serum levels of E2, P, AMH, FSH, and LH in mice of different groups. Serum (**a**) E2, (**b**) P, (**c**) FSH, (**d**) LH, and (**e**) AMH levels. FSH: follicle-stimulating hormone; LH: luteinizing hormone; AMH: anti-Müllerian hormone; E2: estradiol; P: progesterone. All data are expression as mean ± standard deviation mean (‾x ± SD) (*n* = 6). ^★^P < 0.05 and ^★★^*P* < 0.01 compared with the CTX group; ^▲^*P* < 0.05 and ^▲▲^*P* < 0.01 compared with the Con group

We employed enzyme-linked immunosorbent assays (ELISAs) to detect serum FSH, LH, AMH, E2, and P expression levels. The mice in the CTX group had higher FSH and LH levels than those in the control (Con) group, and these levels declined significantly with quercetin intervention (Fig. 1c and d). The CTX group showed decreased AMH, E2, and P expression than those in the Con group. After medium- and high-dose quercetin intervention, AMH, E2, and P levels were all clearly elevated (Fig. 1a, b, and e). These findings suggest that quercetin can increase the expression of sex hormones and AMH in a dose-dependent manner to facilitate functional ovarian recovery.

### Quercetin significantly improved ovarian tissue pathology

**Fig. 2:**
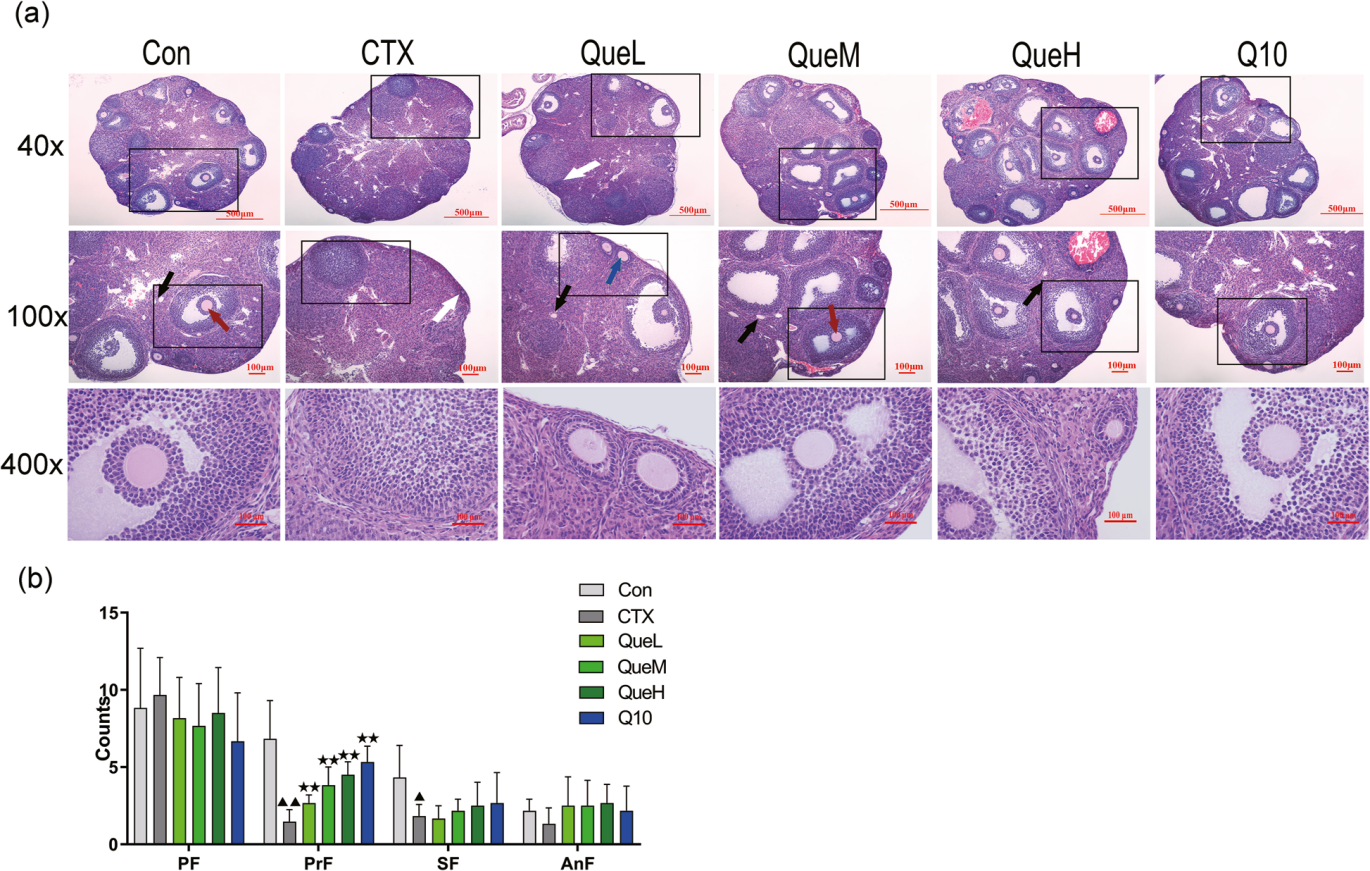
Ovarian tissue pathology assessment in mice of each group. **a** Representative photography by Hematoxylin & Eosin (40 × , 100 × , and 400 ×). The black, blue, red and white arrows indicate primary follicles, secondary follicles, antral follicles and atretic follicles respectively. **b** follicle counting. PF: primordial follicle; PrF: primary follicles; SF: secondary follicles; AnF: antral follicles; AF: atretic follicles. All data are expression as mean ± standard deviation mean (‾x ± SD) (*n* = 6). ^★^*P* < 0.05 and ^★★^*P* < 0.01 compared with the CTX group; ^▲^*P* < 0.05 and ^▲▲^*P* < 0.01 compared with the Con group

After hematoxylin and eosin (H&E) staining, the ovarian tissue morphology was observed under a microscope. The ovaries in the Con group had a large number of corpora lutea and visible follicles at all levels. The ovaries of the CTX group were much smaller than those of the Con group, with fewer primary and secondary follicles, and some atretic follicles. Compared with the CTX group, all the treated groups exhibited an increased in the number of primary follicles. Particularly, in the medium- (QueM) and high-dosage (QueH) quercetin groups, the number of growing follicles at all levels increased, those of atretic follicles decreased, and numerous GCs in mature follicles were neatly arranged in a radial pattern (Fig. 2). These data indicate the potential of quercetin in improving ovarian tissue pathology and increasing the number of ovarian follicles in a dose-dependent manner.

### Quercetin protected against oxidative damage in mice with POI

**Fig. 3:**
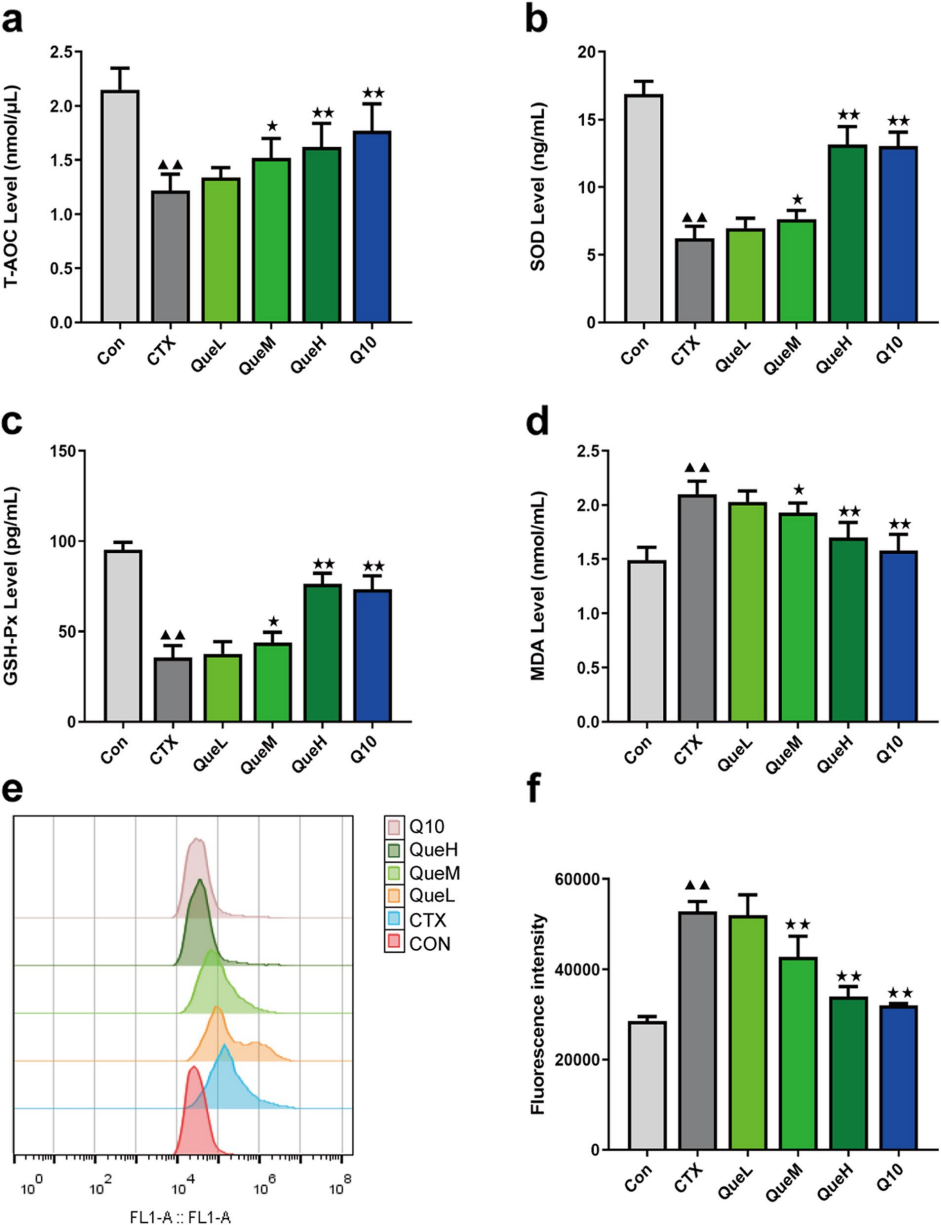
Levels of peroxidation and antioxidant markers in mice of each group. Serum levels of (**a**) T-AOC, (**b**) SOD, (**c**) GSH-Px, (**d**) MDA. **e–f** ROS fluorescence intensity in mouse granulosa cells. T-AOC: total antioxidant capacity; SOD: superoxide dismutase; GSH-Px: glutathione peroxidase; MDA: malondialdehyde; ROS: reactive oxygen species. All data are expression as mean ± standard deviation mean (‾x ± SD) (*n* = 6). ^★^P < 0.05 and ^★★^*P* < 0.01 compared with the CTX group; ^▲^*P* < 0.05 and ^▲▲^*P* < 0.01 compared with the Con group

The levels of total antioxidant capacity (T-AOC), serum superoxide dismutase (SOD), glutathione peroxidase (GSH-Px), MDA, and ROS in each group were assessed to determine the effects of quercetin on oxidative damage. The T-AOC content was substantially lower in the CTX group than in the Con group, and it was elevated in QueM and QueH groups (Fig. 3a). The SOD and GSH-Px contents in the CTX group were the lowest among all the groups. Following quercetin treatment, a dose-dependent increase was observed in GSH-Px content (Fig. 3b and c). Unlike antioxidant enzymes such as SOD and GSH-Px, MDA is the final product of ROS accumulation. As expected, MDA content was higher in the CTX group than in the Con group. Compared with the CTX group, all the treated groups, except the low-dosage quercetin (QueL) group, exhibited a significant decrease in MDA content (Fig. 3d). ROS are crucial products of oxidative stress. Maximum ROS production was detected in the CTX group, whereas mitochondrial ROS production was notably decreased in the QueM and QueH groups (Fig. 3e). These findings demonstrate the ability of quercetin to increase T-AOC, SOD, and GSH-Px levels and decrease MDA and ROS levels in the ovarian tissues, implying that it can function as an antioxidant in mice with POI.

### Quercetin enhanced mitochondrial function in the GCs of POI mice

**Fig. 4:**
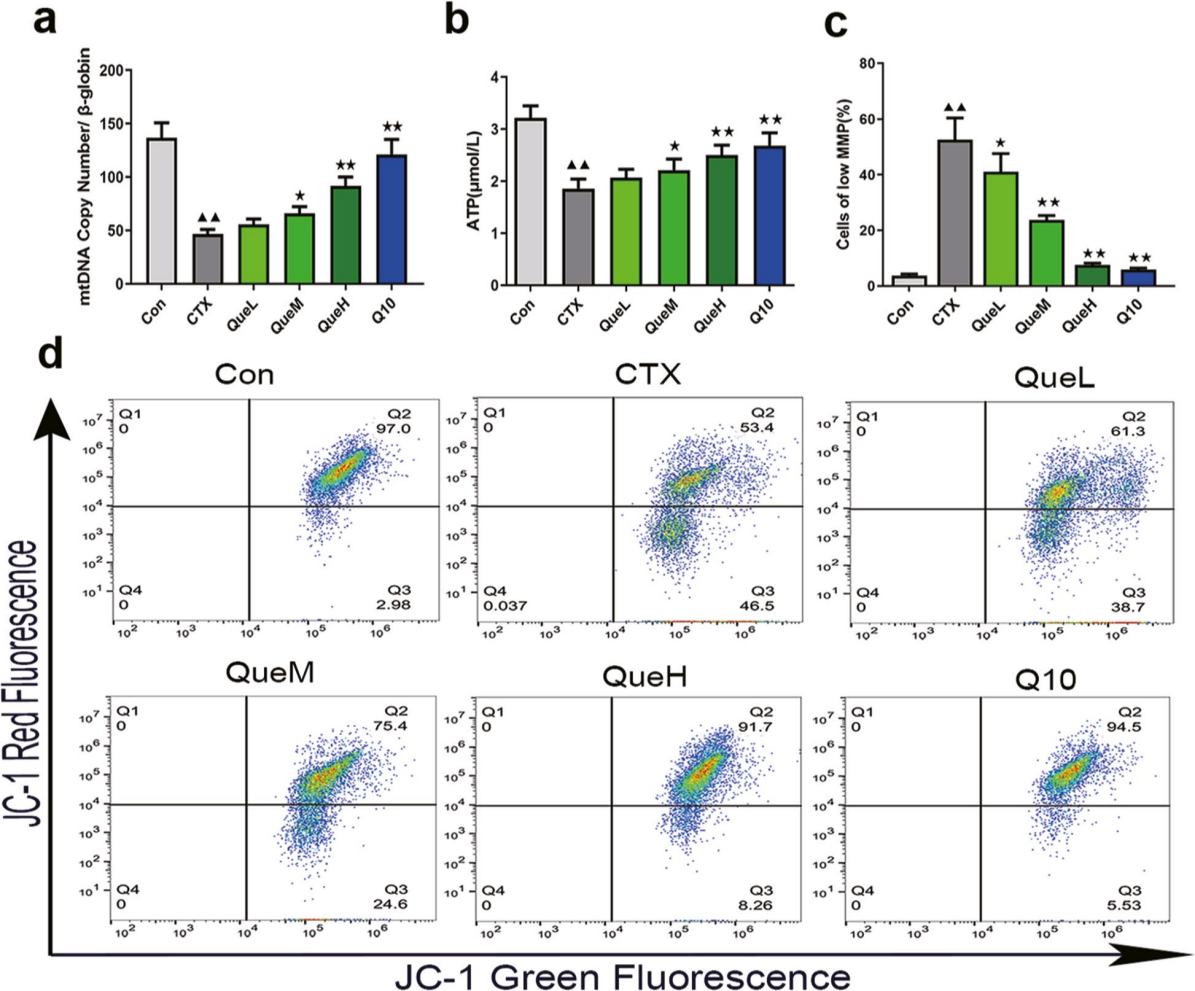
Mitochondrial function assessment in ovarian granulosa cells of each group. Levels of (**a**) relative mtDNA copy number and (**b**) ATP expression in different groups; **c–d** Degradation of MMP. mtDNA: mitochondrial DNA; ATP: Adenosine triphosphate.; MMP: mitochondrial membrane potential. All data are expression as mean ± standard deviation mean (‾x ± SD) (n = 3). ^★^*P* < 0.05 and ^★★^*P* < 0.01 compared with the CTX group; ^▲^*P* < 0.05 and ^▲▲^*P* < 0.01 compared with the Con group

To further explore the antioxidant mechanism of quercetin, we measured mtDNA production, ATP production, and mitochondrial membrane potential (MMP) levels, which are effective indicators of mitochondrial function. The number of mtDNA copies in the CTX group was significantly reduced. The mtDNA copy levels increased to different extents in all the intervention groups, of which the QueH and coenzyme 10-treated (Q10) groups displayed the best effect (Fig. 4a). Compared with the Con group, ATP production in the GCs of the CTX group was significantly reduced. Following quercetin treatment, ATP production increased in a dose-dependent manner compared to the CTX group (Fig. 4b). The CTX group had a higher rate of MMP reduction than the Con group (Fig. 4c). Following quercetin intervention, the results showed a dose-dependent decrease in the MMP reduction rate compared to the Con group (Fig. 4c and d). In addition, we evaluated the morphology of the mitochondria in the GCs of each group. Most of the mitochondria in the Con group were well-developed, and the crest structures were visible. In the CTX group, severe mitochondrial edema and cristae appeared broken or were non-existent. The mitochondrial edema in the QueM and QueH groups was reduced, with minor structural disorders in mitochondrial cristae. These findings highlight the ability of quercetin to reduce MMP reduction rate and increase mtDNA synthesis and ATP production in the GCs of POI mice, resulting in an improved mitochondrial function.

### Quercetin improved mitochondrial redistribution in the GCs of POI mice

**Fig. 5:**
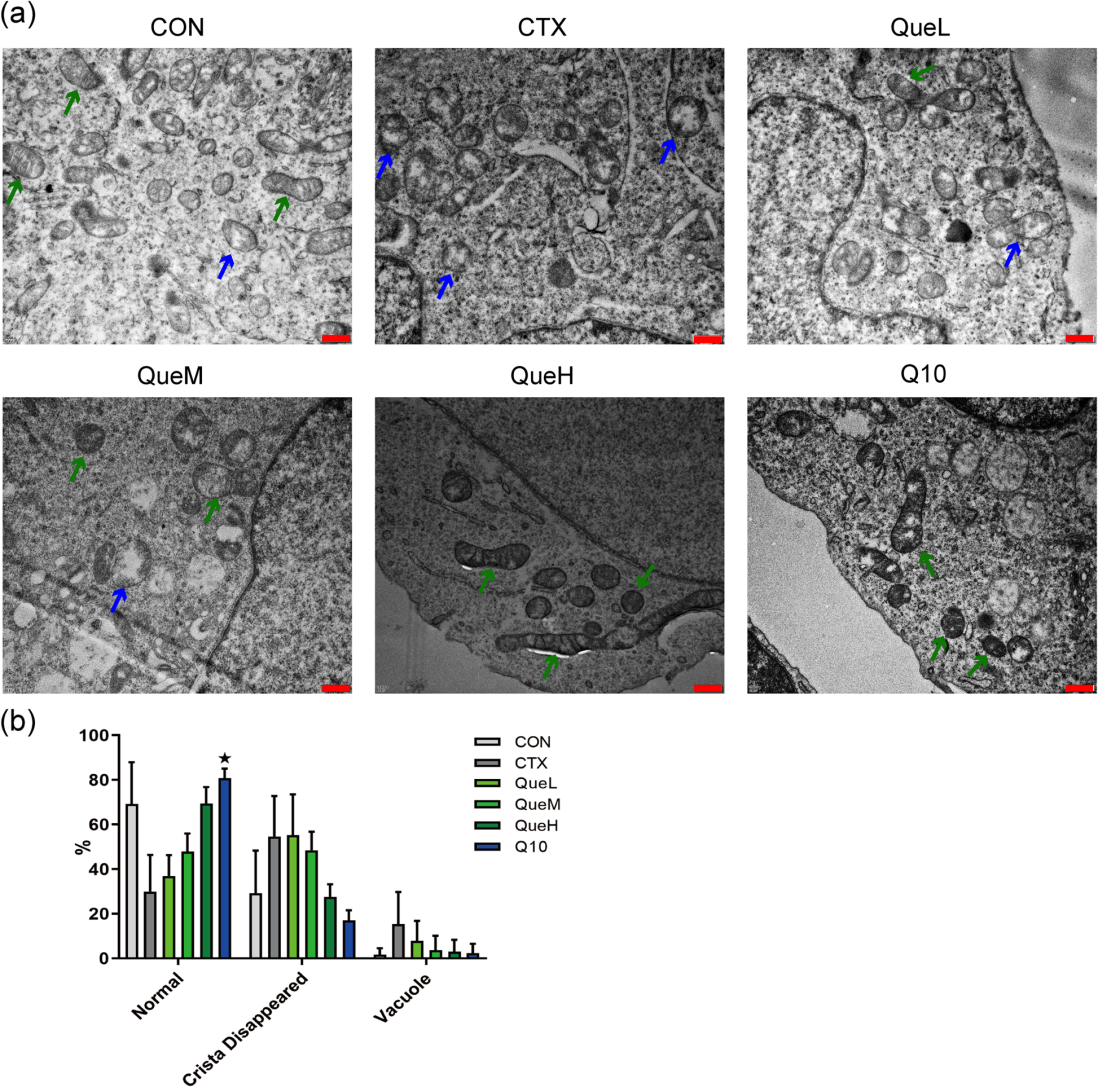
Mitochondrial ultrastructure under the transmission electron microscopy in mouse granulosa cells of each group. **a** transmission electron microscopy characterization of mitochondria. Green and blue arrows indicate normal mitochondria, irregular mitochondria respectively. Scale bar: 500 nm. **b** percentage normal and abnormal mitochondria. All data are expression as mean ± standard deviation mean (‾x ± SD) (n = 3). ^★^*P* < 0.05 compared with the CTX group

The mitochondria in the Con group were well-developed; the cristae were clearly visible and aggregated and were distributed near the nucleus. However, the mitochondria in the CTX group were swollen compared to those in the Con group, with mitochondrial cristae disappearing or becoming vacuolated. The number of intracellular vacuolated mitochondria was reduced and the number of normal mitochondria were increased in all the quercetin and Q10 groups (Fig. 5). These findings indicate that quercetin can improve mitochondrial redistribution, leading to improved mitochondrial activity.

### Quercetin upregulated peroxisome proliferator-activated receptor gamma coactivator 1-alpha (PGC1α), mitochondrial transcription factor A (TFAM), and SOD2 expression at the mRNA and protein levels in the GCs of POI mice

**Fig. 6:**
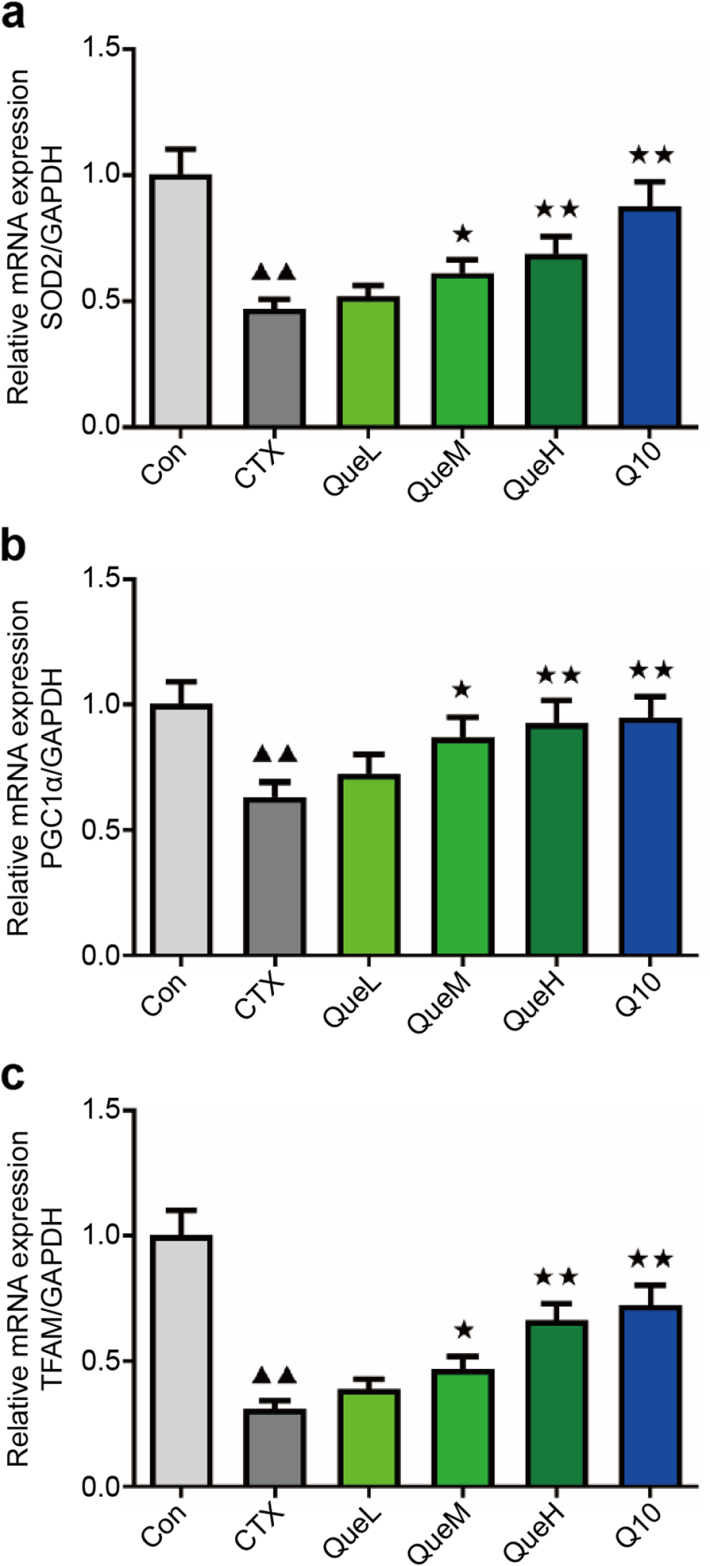
PGC1α, TFAM, and SOD2 mRNA expression in granulosa cells of each group. mRNA expression of (**a**) SOD2, (**b**) PGC1-α, and (**c**) TFAM. PGC1-α: peroxisome proliferator-activated receptor gamma coactivator 1-alpha TFAM: mitochondrial transcription factor A; SOD2: superoxide dismutase 2. All data are expression as mean ± standard deviation mean (‾x ± SD) (n = 3). ^★^*P* < 0.05 and ^★★^*P* < 0.01 compared with the CTX group; ^▲^*P* < 0.05 and ^▲▲^*P* < 0.01 compared with the Con group (*n* = 3)

**Fig. 7:**
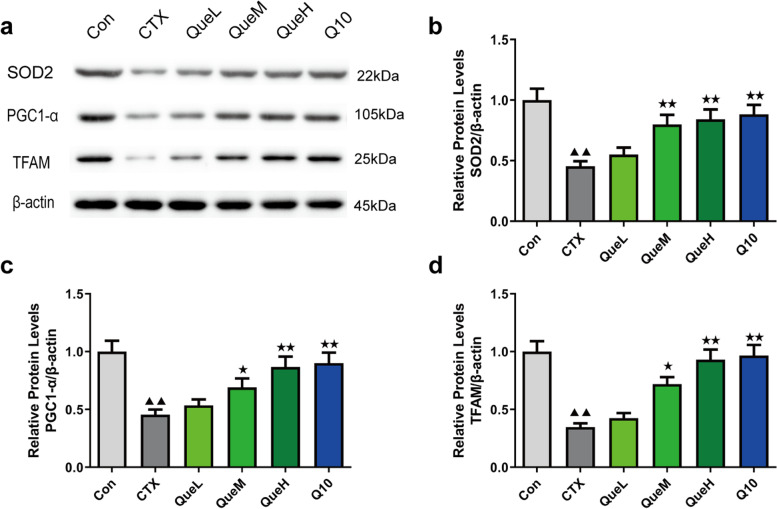
PGC1α, TFAM, and SOD2 protein expression in granulosa cells of each group. Original blots/gels are provided separately. **a** protein band, (**b**) protein expression of SOD2, (**c**) protein expression of PGC-1α, and (**d**) protein expression of TFAM. PGC1α: peroxisome proliferator-activated receptor gamma coactivator 1-alpha TFAM: mitochondrial transcription factor A; SOD2: superoxide dismutase 2. All data are expression as mean ± standard deviation mean (‾x ± SD) (n = 3). ^★^*P* < 0.05 and ^★★^*P* < 0.01 compared with the CTX group; ^▲^*P* < 0.05 and ^▲▲^*P* < 0.01 compared with the Con group

The mRNA and protein levels of TFAM, PGC1α, and SOD2 in the ovarian GCs of mice were lower in the CTX group than in the Con group (Figs. 6 and 7). Moreover, the mRNA and protein levels of TFAM, PGC1, and SOD2 were considerably higher in the QueM, QueH, and Q10 groups than in the CTX group (Figs. 6 and 7). These data demonstrate the ability of quercetin to promote the mRNA and protein levels of TFAM, PGC1α, and SOD2, leading to the improved mitochondrial function of GCs.

### Quercetin reduced the protein expression of NLRP3, gasdermin D (GSDMD), caspase-1, and IL-1β in the GCs of POI mice

**Fig. 8:**
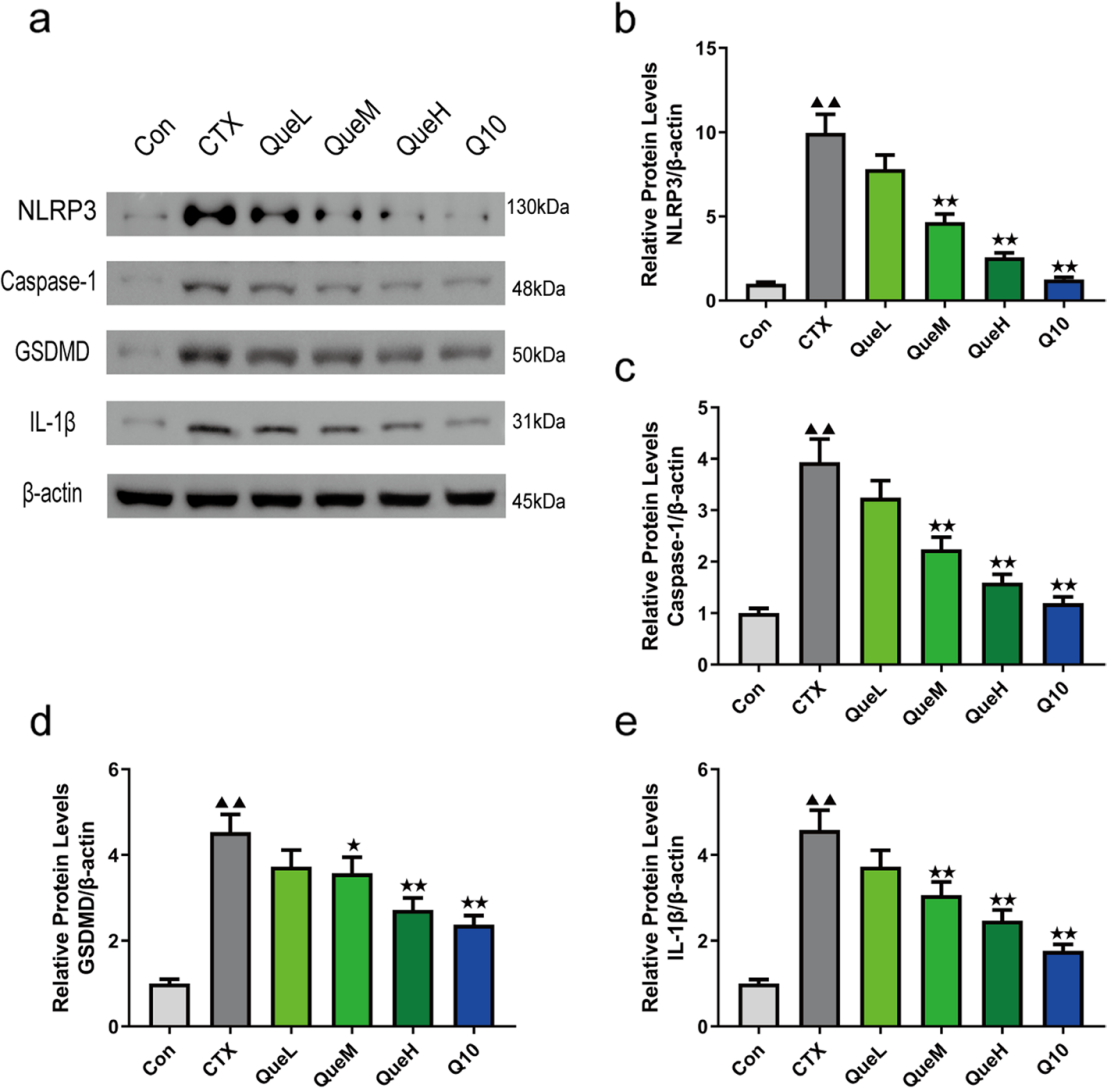
NLRP3, caspase-1, GSDMD, and IL-1β protein expression in granulosa cells of each group. Original blots/gels are provided separately. **a** protein band, (**b**) protein expression of NLRP3, (**c**) protein expression of Caspase-1, (**d**) protein expression of GSDMD, and (**e**) protein expression of IL-1β. NLRP3: NOD-like receptor pyrin domain containing 3; GSDMD: gasdermin D. All data are expression as mean ± standard deviation mean (‾x ± SD) (*n* = 3). ^★^*P* < 0.05 and ^★★^*P* < 0.01 compared with the CTX group; ^▲^*P* < 0.05 and ^▲▲^*P* < 0.01 compared with the Con group

To confirm that pyroptosis is involved in POI progression, we examined NLRP3, GSDMD, caspase-1, and IL-1β expression. The results revealed that compared with the Con group, the CTX group had considerably higher levels of these proteins, and treatment with quercetin and coenzyme Q10 reduced the levels of these proteins (Fig. 8). These findings indicate that quercetin inhibits pyroptosis by diminishing NLRP3, GSDMD, caspase-1, and IL-1β protein expression in the GCs of POI mice.

## Discussion

The findings of the present study indicate that quercetin supplementation protects the ovarian reserve from CTX-induced ovarian damage, resulting in the elevation of serum AMH, E2, and P levels, reduction of FSH and LH expression, and alleviation of ovarian pathology. Further, quercetin displayed a protective function, reflected by the increase in ATP levels, mtDNA copy number, and MMP, and the upregulation of PGC1α, SOD2, and TFAM at both the mRNA and protein levels. In addition, quercetin may exert an anti-pyroptosis potential by suppressing NLRP3, caspase-1, IL-1β, and GSDMD levels in the GCs of POI mice.

We first established a POI mouse model by injecting CTX (90 mg/kg dose), Various dosages and procedures have been used to induce POI in animals [28, 29]. CTX is the most commonly used chemotherapeutic drug. The ovary, in particular, is negatively affected by CTX in female reproductive organs. Its hazardous side effects are linked to its active metabolites, which interfere with ovarian antioxidant defense mechanisms and cause ROS via conjugating with glutathione to disrupt DNA synthesis [30]. According to our results, CTX can lead to a significant decrease in the number of both the primary and secondary follicles, and the ovarian volume in the model mice was smaller than that in the control mice. These results were consistent with those of a previous study that established a POI model by CTX injection [31–34]. AMH is the most accurate biomarker of ovarian aging [35] and is primarily secreted by ovarian GCs. FSH and LH are crucial hormones for ovarian development, and the FSH/LH ratio is regarded as an independent factor for predicting pre-POI and early ovarian decline [36]. In addition to its well-known roles, FSH also regulates the expression of E2 and P, both of which act as important markers for ovarian GC maturation [37]. Thus, they can also be utilized as ovarian reserve markers.

In the present study, the serum AMH, E2, and P levels in the model group were significantly decreased. Simultaneously, morphological degradation of the ovarian tissue was observed under a light microscope after H&E staining. Following quercetin administration for 28 days, the AMH, E2, and P levels were significantly elevated, accompanied by a reduction in the degradation of follicles at all stages. Increases in the levels of ovarian reserve markers and morphological changes suggest that quercetin has a favorable effect on rebuilding ovarian function following CTX exposure.

GCs are the main source of steroid hormones including E2 and P [38], then they influence FSH and LH via feedback regulation. Hence, we believe that viability of GCs is the key role of the effect of quercetin on production or inhibition of sex-related hormones. Quercetin is indeed confirmed that affect the proliferation and the apoptosis of GCs [38]. To further explore the underlying mechanisms we went to investigate the effect of quercetin on GCs from the perspective of OS and pyroptosis.

Accumulating evidence has revealed the significance of ROS in triggering mitochondrial dysfunction. After POI progression, excessive ROS causes the oxidative damage of proteins, nucleic acids, and lipids, ultimately leading to cell death [39]. MDA is the final lipid peroxidation product [40] and T-AOC refers to the total antioxidative capacity. In contrast, SOD, along with GSH-Px, is an effective ROS scavenger. Increased levels of ROS reflect the ability to resist oxidative damage [41]. In the induced POI model, the expression of oxidative stress products (ROS, T-AOC, and MDA) increased, whereas that of antioxidant products (SOD and GSH-Px) decreased. After quercetin treatment, the oxidative stress levels dramatically declined. High doses of quercetin, especially 50 mg/kg, exerted a protective effect against oxidative stress by exhibiting antioxidant abilities in the CTX-induced ovarian damage model, which was consistent with previous research [42].

The represents the quantity and quality of ovarian follicle composed of GCs and oocytes [43]. The role of mitochondria in ovarian reserve is due primarily to ATP production, which is vital for ovarian cellular function. [44]. mtDNA mutation is the most common biomarkers for mitochondrial dysfunction. clinical researches suggested that mtDNA content in GCs was related to oocyte quality, and mtDNA mutation play a promoting role in the progression and pathogensis of POI [45, 46]. Moreover, improvement mitochondrial function through supplementation of components of the respiratory chains is considered as a feasible method for recover ovarian reserve [47].

The mitochondria are a significant site for ROS production. Excessive ROS accumulation can damage the mitochondria [48]. Mitochondrial ATP production, MMP, and mtDNA production levels are substantial indicators of mitochondrial function. Multiple studies have shown that excessive ROS damages mtDNA, downregulates MMP, and reduces intracellular ATP levels [49, 50]. Consistent with these findings, our results showed that quercetin reversed the affected mitochondrial functions by increasing ATP and mtDNA production and decreasing the MMP reduction rate. To further investigate mitochondrial oxidative damage, we used transmission electron microscopy (TEM) to determine the mitochondrial ultrastructure. We observed that the decrease in the number of normal mitochondria induced by CTX was reversed by quercetin treatment. These results suggested that quercetin exerts protective effects by enhancing mitochondrial functions to prevent CTX-induced mitochondrial damage. Mitochondrial biogenesis is a complex biological process involving the formation of mitochondrial membranes and the synthesis of mitochondrial proteins and mtDNA [51]. Several studies have shown that quercetin could bolster mitochondrial biogenesis in mammalian cells [52]. PGC1-α is known as the “master regulator” and is involved in mitochondrial biogenesis; it interacts with the nuclear respiratory factors (NRF) (e.i.NRF-1, NRF-2), initiates NRF transcription, followed by the activation of mitochondrial transcription factor A (TFAM). TFAM is crucial for the replication of mtDNA and the transcription of mitochondria-encoded genes, resulting in mitochondrial generation [53–55]. The close relationship between PGC1-α and OS resistance has been indicated by diverse studies in the liver, brain, muscle tissue, or cell lines [56–59]. A few studies have reported that quercetin promotes mitochondrial biogenesis in GCs. Thus, we speculated that quercetin might suppress ovarian damage via the PGC1-α pathway. The results analyzed using western blotting and RT-qPCR supported our speculation that quercetin increases the expression of PGC1-α, TFAM, and SOD2 in the QueM and QueH groups.

Recent evidence has demonstrated the role of mitochondrial ROS in inflammasome activation [60, 61]. ROS could act like the “kindling” or triggering factor to activate NLRP3 inflammasomes, resulting in inflammatory pathological processes [19]. The NLRP3 inflammasome is a multiprotein complex formed by apoptosis-associated speck-like protein (ASC), NLRP3, and procaspase-1 [62]. Activation of NLRP3 facilitates the induction of the downstream effector caspase-1, which subsequently triggers the excessive release of IL-1β and IL-18, ultimately initiating programmed pyroptosis. As a rapid form of host cell death, pyroptosis is involved in the progression of various diseases that are characterized by cell swelling, membrane pore formation, membrane rupture, and the extensive release of inflammatory cytokines [63–66].

Based on the existing finding of excessive ROS expression in the POI model resulting in ovarian damage, we hypothesized that pyroptosis might occur in the ovarian tissue after the CTX injection. We detected the protein expression of inflammasome components (NLRP3, caspase-1, IL-1β) as well as GSDMD. Our results indicated that the quantities of these pyroptosis-related proteins were considerably higher in the CTX group than those in the control. Treatment with quercetin and coenzyme Q10 decreased the expression of these proteins, which is consistent with our previous hypothesis that quercetin protects against ovarian ROS damage through the anti-pyroptosis pathway, in addition to its antioxidant activity.

It is worth pointing out that autophagy may be the intermediate link connecting ROS and pyroptosis [67]. ROS accumulation and mitochondrial dysfunction can trigger autophagy/mitophagy that clear up damaged mitochondria and controlling mitochondrial mass [68]. First, ROS is an important activating factor for NLRP3 inflammasome, so autophagy can reduce NLRP3-dependent pyroptosis by the clearance of ROS in damaged mitochrondria [67]. Moreover, various autophagy-related proteins can inhibit secretion of IL-1β and cell lysis in pyroptosis[69]. Research found that blocked autophagy enhanced the activation of the NLRP3-dependent pyroptosis in chronic cerebral hypoperfusion [70], but the relationship between ROS, autophagy, and pyroptosis in POI is still unclear and worthy to be explored in our future studies.

Interestingly, coenzyme Q10 showed an effect similar to that of high-dose quercetin. This may be because coenzyme Q10 plays a crucial role as an electron carrier inside the mitochondrial electron transport chain [71] and directly mediates the expression of several genes inside the mitochondria, including those involved in inflammation [67, 72]. This also illustrates the excellent protective capacity of quercetin against oxidative stress and mitochondrial ability from another perspective. The main limitation of the present study is the lack of in vitro experiments to validate the present results; hence, further in vitro investigation is needed in the future.

## Conclusions

In summary, we demonstrated that mitochondrial dysfunction contributes to POI pathogenesis. Quercetin administration reverses mitochondrial dysfunction by inducing antioxidant damage and activating mitochondrial biogenesis through the PGC1-α pathway. Moreover, our study proved our hypothesis that quercetin protects ovarian function by downregulating pyroptosis in a CTX-induced POI model. These results indicate that quercetin can be considered a potential agent for treating POI.

## Methods

### Animals

C57BL/6 female mice (6–8 weeks old) were purchased from the Shanghai SLAC Laboratory Animal Company (Certificate No. SCXK 2013–0018, Shanghai, China) and housed in the barrier system of the Animal Experiment Center, located in Zhejiang Chinese Medical University, under pathogen-free conditions, 22 ± 2 °C, and 12 h light/dark cycle with lights on at 7:00 a.m. The Institutional Animal Care and the Animal Experiment Center of the Zhejiang Chinese Medical University approved all the experimental procedures used in this study (approval number: IACUC-20180402–02).

### Experimental grouping and model establishment

After an acclimatization period (5–7 days), a 10-day estrus cycle detection was implemented to exclude mice with abnormal ovarian functions. Then, 36 female mice were randomly and equally distributed into six groups: A: Con (control group); B: CTX (CTX model group); C: QueL (low-dosage quercetin group, 12.5 mg/kg); D: QueM (medium-dosage quercetin group, 25 mg/kg); E: QueH (high-dosage quercetin group, 50 mg/kg); F: Q10 (Coenzyme Q10 group, 1.25 mg/kg). The mice in the Con group received a single intraperitoneal injection of 200 μL saline, while the others were administered an equal volume of saline containing 90 mg/kg CTX (GC111451, Glpbio, Montclair, USA), a dose reported to lead to an ovarian immune disturbance that causes diminished ovarian reserve in mice [73]. After one-time intraperitoneal injection of CTX with a dose of 90 mg/kg, vaginal exfoliated cell smears were performed at 8:00 a.m. daily for two weeks, and the model establishment was regarded as successful if the irregular estrous cycle was observed. The Con and CTX groups were intragastrically administered normal saline. In contrast, the quercetin groups were administered various concentrations of quercetin solution (12.5–50 mg/kg) at 8:00 a.m., the doses of quercetin were determined based on previous studies [74, 75], and the Q10 group was perfused with 1.25 mg/kg coenzyme Q10. After four consecutive weeks, the mice were sacrificed by CO_2_ exposure 48 h after the administration of 10 IU of pregnant mare serum gonadotropin.

### Extraction of primary ovarian GCs

After the mice were sacrificed, the ovaries of the mice were washed with phosphate-buffered saline (PBS), and the surrounding fat tissue was removed; F12 medium was added to the petri dish, and the follicles were punctured using a sterile 1-mL syringe needle to release the GCs into the medium. The cells in the Pasteur tube were gently blown and centrifuged at 100 × *g* for 3 min after they were filtered using a 200-mesh cell sieve. The supernatant was discarded, the complete medium was added and the cells were transferred to a new sterile petri dish and then placed in a 37 °C incubator for cultivation at 5% CO_2_.

### ELISA

SOD, GSH-Px, T-AOC, MDA, E2, FSH, LH, P, and AMH levels were detected using specific ELISA kits (Maiman, Jiangsu, China) according to the manufacturer’s instructions.

### Histopathology and ultrastructural morphology evaluations

After the mice were sacrificed, the ovary tissues were removed and fixed in 4% paraformaldehyde, followed by dehydration, paraffin embedding, and serial sectioning. Ovary sections with a thickness of 4 μm were obtained and stained with H&E on glass slides. Images of the ovary structure and follicle counts were obtained using a microscope (Nikon Eclipse E100). For observing the mitochondrial morphology, immediately after dissection, the ovary tissue was fixed with glutaraldehyde and osmic acid, dehydrated through a series of graded ethanol solutions, and then ingrained in a resin mixture. Ultrathin sections were cut to a thickness of 100 nm and stained with lead citrate and uranyl acetate. TEM images of mitochondrial morphology were obtained using a Hitachi 7650 TEM instrument.

### Assessment of ROS levels

The six-well plate was filled with 10 M 2′-7′ dichlorofluorescin diacetate to completely cover the GCs, which were subsequently incubated for 20 min at 37 °C in a cell incubator. The GCs were washed and suspended in 100 μL of PBS, and the fluorescence intensity of ROS was detected by flow cytometry (BD FACSVerse, BD Biosciences).

### Assessment of MMP

To assess changes in the MMP, JC‐1 (Beyotime Institute of Biotechnology, Shanghai, China) was used as a fluorescent probe. GCs were collected and stained with JC‐1 staining solution for 30 min at 37 °C, and flow cytometry (BD FACSVerse, BD Biosciences) was used to evaluate the samples.

### Quantification of ATP levels

The chemiluminescence ATP assay kit was used as per the manufacturer’s instructions (Beyotime Institute of Biotechnology). The reagents to be used in an ice bath were melted, the ATP standard solution was diluted with ATP detection lysate to several concentrations of 0.1, 0.3, 1, 3, and 10 µM, and the corresponding standard curve was drawn. Subsequently, 100 µL of ATP detection working solution was added into the detection hole at 20–25 °C. Subsequently, 20 µL of the standard or sample was immediately added to each well and mixed. After at least 2 s, the relative light unit value was obtained using a Glomax 20/20 luminometer (Promega Corporation, Madison, WI, USA) and a standard curve was used to compute the concentration of ATP.

### Quantitative real-time PCR

Quantitative real-time PCR was performed to detect the mtDNA copy number and the content of SOD2, PGC1-α, and TFAM. Total RNA was extracted from the GCs and reverse-transcribed into cDNA. SYBR Premix Ex TaqTM TM II (Perfect Real Time) (TaKaRa, Shiga, Japan) was used to detect the expression of the specified genes. The following conditions were used for reverse transcription: 42 °C for 15 min, 85 °C for 5 s, cooling to 4 °C for 10 min, and refrigeration at − 20 °C. The reverse transcription reaction mixture was added to the tubes used for quantitative fluorescence reaction, and the amplification reaction conditions were as follows: pre-denaturation at 95 °C for 10 min, followed by 40 cycles (95 °C for 15 s and 60 °C for 60 s). The results of the reaction were collected, and normalized expression was calculated as the relative fold change using the 2 ^− ΔΔCT^ method. Table 1 shows the primer sequences used for quantitative real-time PCR of the genes. GAPDH was used as the internal reference gene.

**Table 1:** Primer sequences

Gene	Forward primer (5′-3′)	Reverse primer (3′-5′)
Mouse TFAM	AACACCCAGATGCAAAACTTTCA	GACTTGGAGTTAGCTGCTCTTT
Mouse PGC1α	GACAGCTTTCTGGGTGGATTG	GGAGACTGGGCCGTTTAGT
Mouse SOD2	CAGACCTGCCTTACGACTATGG	CTCGGTGGCGTTGAGATTGTT
Mouse mtDNA	AACCTGGCACTGAGTCACCA	GGGTCTGAGTGTATATATCATGAAGAGAAT
Mouse GAPDH	TCAACGGCACAGTCAAGG	TGAGCCCTTCCACGATG
Mouse β-globin	CCCTTGGACCCAGAGGTTCT	AGTACCGTTCTTTCACGAGC

### Western blotting

The GCs were lysed on ice in radioimmunoprecipitation buffer (Beyotime Institute of Biotechnology), and the protein content was determined using the bicinchoninic acid Protein Assay Kit (Solarbio, Beijing, China) on a 4–12% gel subjected to sodium dodecyl sulfate–polyacrylamide gel electrophoresis (Biosharp, Beijing, China). After electrophoresis and transfer to polyvinylidene difluoride membranes, the membranes were blocked with bovine serum albumin and incubated overnight at 4 °C with primary antibodies against TFAM, PGC1α, SOD2, NLRP3, GSDMD, IL-1β, and caspase-1 [64] (Cell Signaling Technology, Danvers, MA, USA) diluted at 1:1000. β-Actin (1:1000, Cell Signaling Technology) was used as the internal reference. The membranes were washed thrice with Tris-buffered saline with Tween-20 (TBST), and the blots were detected using an enhanced chemiluminescence (ECL) substrate system after a 1-h incubation with secondary antibodies (Cell Signaling Technology). The blots were analyzed using ImageJ 1.8.0.

### Statistical analyses

All experiments were replicated more than three times, and the data are reported as the mean ± standard deviation (SD). All groups were analyzed for multiple comparisons by one-way analysis of variance (ANOVA) using Prism 8.0.2. Additionally, statistical significance was set at *P* < 0.05 and *P* < 0.01.

## Data Availability

The datasets used and/or analyzed during the current study are available from the corresponding author (zhaqin01@163.com) on reasonable request.

## Abbreviations

CTX: Cyclophosphamide
POI: Primary ovarian insufficiency
ELISA: Enzyme-linked immunosorbent assay
MMP: Mitochondrial membrane potential
PGC1α: Peroxisome proliferator-activated receptor gamma coactivator 1-alpha
TFAM: Mitochondrial transcription factor A
SOD: Superoxide dismutase
NLRP3: NOD-like receptor pyrin domain containing 3
GSDMD: Gasdermin D
GC: Granulosa cell
OS: Oxidative stress
ROS: Reactive oxygen species
mtDNA: Mitochondrial DNA
IL-1β: Interleukin-1β
PBS: Phosphate-buffered saline
GSH-Px: Glutathione peroxidase
T-AOC: Total antioxidant capacity
MDA: Malondialdehyde
E2: Estradiol
FSH: Follicle-stimulating hormone
LH: Luteinizing hormone
P: Progesterone
AMH: Anti-Müllerian hormone
H&E: Hematoxylin and eosin
TEM: Transmission electron microscopy
TBST: Tris-buffered saline with Tween-20
SD: Standard deviation
ANOVA: One-way analysis of variance
